# Supplementary material for: Phenotypic Characterization and Draft Genome Sequence Analyses of Two Novel Endospore-Forming Sporosarcina spp. Isolated from Canada Goose (Branta canadensis) Feces
Source: Microorganisms. 2023 Dec 29;12(1):70. doi: 10.3390/microorganisms12010070 (PMC10818898; doi:10.3390/microorganisms12010070)
Supplement: Supplementary file 1 [file microorganisms-12-00070-s001.zip › Keshri et al. Figure S2.pdf]

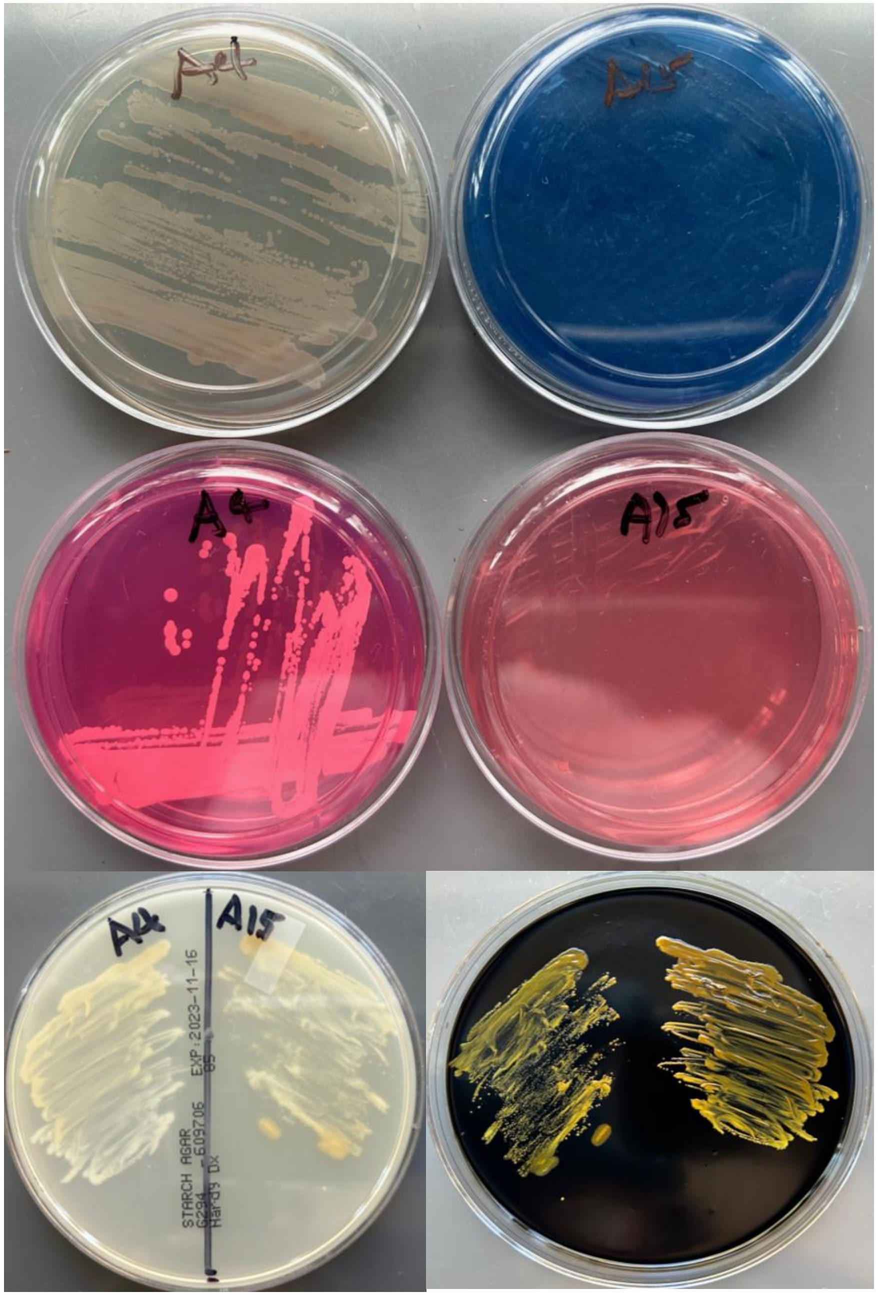

Fig. S2. Growth media analyses of Sporosarcina isolates A4 (left side) and A15 (right side). Upper plates A4 and A15 on spirit blue agar, middle plates on mannitol salt agar and bottom plates on starch plates. Starch plates on the right side are stained with Gram iodine.

Methods: The A4 and A15 isolates were plated on mannitol salt agar [27] media. The isolates were also assayed for lipase activity using spirit blue agar plates [30] and for starch hydrolysis [31].
