# Supplementary material for: Phenotypic Characterization and Draft Genome Sequence Analyses of Two Novel Endospore-Forming Sporosarcina spp. Isolated from Canada Goose (Branta canadensis) Feces
Source: Microorganisms. 2023 Dec 29;12(1):70. doi: 10.3390/microorganisms12010070 (PMC10818898; doi:10.3390/microorganisms12010070)
Supplement: Supplementary file 1 [file microorganisms-12-00070-s001.zip › Keshri et al. Table S1.pdf]

Table S1. Metabolic profiling of strains A4 and A15 on Biolog PM9 and PM10 MicroPlate.

PM9 Osmolytes	Strain A4	Strain A15
NaCl 1%	++	++
NaCl 2%	++	++
NaCl 3%	++	++
NaCl 4%	+	+
NaCl 5%	+	−
NaCl 9%	+	−
NaCl 10%	+	−
NaCl 6% + Creatine	+	−
NaCl 6% + Creatinine	+	−
NaCl 6% + KCl	++	+
NaCl 6% + L-Proline	+	−
NaC1 6% + β-Glutamic Acid	+	−
NaC1 6% + γ–Amino -N- Butyric Acid	+	−
NaC1 6% + Glutathione	+	+
NaC1 6% + Glycerol	+	−
NaC1 6% + Trehalose	++	−
NaC1 6% + Trimethylamine- N-oxide	+	−
NaC1 6% + Trimethylamine	+	−
NaC1 6% + Trigonelline	+	−
Potassium chloride 3%	+	++
Potassium chloride 4%	+	++
Potassium chloride 5%	−	++
Potassium chloride 6%	−	++
Sodium sulfate 2%	+	++
Sodium sulfate 3%	++	++
Sodium sulfate 4%	+	++
Sodium sulfate 5%	++	++
Ethylene glycol 5%	−	++
Ethylene glycol 10%	−	++
Sodium formate 1%	−	++
Urea 2%	−	++
Sodium Lactate 1%	−	++
Sodium Phosphate pH 7 20mM	−	++
Sodium Phosphate pH 7 -50mM	−	++
Sodium Phosphate pH 7 -100mM	−	+
Sodium Phosphate pH 7 -200mM	+	−
Ammonium sulfate pH8 -10mM	+	++
Ammonium sulfate pH8 -20mM	+	++

Ammonium sulfate pH8 -50mM	−	++
Ammonium sulfate pH8 -100mM	−	++
Sodium Nitrate 10mM	−	++
Sodium Nitrate 20mM	+	++
Sodium Nitrate 40mM	+	++
Sodium Nitrate 60mM	+	++
Sodium Nitrate 80mM	+	++
Sodium Nitrate 100mM	+	++
Sodium Nitrite 10mM	−	++
Sodium Nitrite 20mM	−	++
Sodium Nitrite 40mM	−	++
Sodium Nitrite 60mM	−	+
**PM10 pH**		
pH 6	−	++
pH 7	−	++
pH 8	+	++
pH 8.5	+	++
pH 9	+	++
pH 9.5	++	++
pH 10	++	+
pH 9.5	+	+
pH 9.5 + L-Alanine	++	+
pH 9.5 + L-Arginine	++	++
pH 9.5 + L-Asparagine	++	++
pH 9.5 + L-Aspartic Acid	++	++
pH 9.5 + L-Glutamic Acid	++	++
pH 9.5 + L-Glutamine	++	++
pH 9.5 + Glycine	++	++
pH 9.5 + L-Histidine	++	++
pH 9.5 + L-Isoleucine	++	++
pH 9.5 + L-Leucine	++	++
pH 9.5 + L-Lysine	++	+
pH 9.5 + L-Methionine	++	++
pH 9.5 + L-Phenylalanine	++	++
pH 9.5 + L-Proline	++	++
pH 9.5 + L-Serine	++	++
pH 9.5 + L-Threonine	++	++
pH 9.5 + L-Tryptophan	++	++
pH 9.5 + L-Tyrosine	++	++

pH 9.5 + L-Valine	++	++
pH 9.5 + Hydroxy- L-Proline	++	++
pH 9.5 + L-Ornithine	++	++
pH 9.5 + L-Homoarginine	++	++
pH 9.5 + L-Homoserine	++	++
pH 9.5 + Anthranilic acid	+	++
pH 9.5 + L-Norleucine	++	++
pH 9.5 + L-Norvaline	++	++
pH 9.5 + Agmatine	+	+
pH 9.5 + Cadaverine	++	++
pH 9.5 + Putrescine	++	++
pH 9.5 + Histamine	++	++
pH 9.5 + Phenylethylamine	+	+
pH 9.5 + Tyramine	+	+
pH 9.5 + Creatine	+	+
pH 9.5 + Trimethylamine- N-oxide	+	+
pH 9.5 + Urea	++	++
X–α-D-Glucoside	+	++
X-β-D-Glucoside	+	++
X-α-D-Galactoside	+	++
X-β-D-Galactoside	−	++
X-α- D-Glucuronide	−	++
X-β- D-Glucuronide	−	+
X-β-D-Galactosaminide	−	+
X-α-D-Mannoside	−	++
X-PO4	−	+
X-SO4	+	++

"+" and "−" indicates the ability (or not) of the strain to actively grow and reduce the redox dye present in the well, in presence of the different stresses: "++" strong positive, "+" positive, "−" negative.
