# Supplementary material for: Phenotypic Characterization and Draft Genome Sequence Analyses of Two Novel Endospore-Forming Sporosarcina spp. Isolated from Canada Goose (Branta canadensis) Feces
Source: Microorganisms. 2023 Dec 29;12(1):70. doi: 10.3390/microorganisms12010070 (PMC10818898; doi:10.3390/microorganisms12010070)
Supplement: Supplementary file 1 [file microorganisms-12-00070-s001.zip › Keshri et al. Figure S1.pdf]

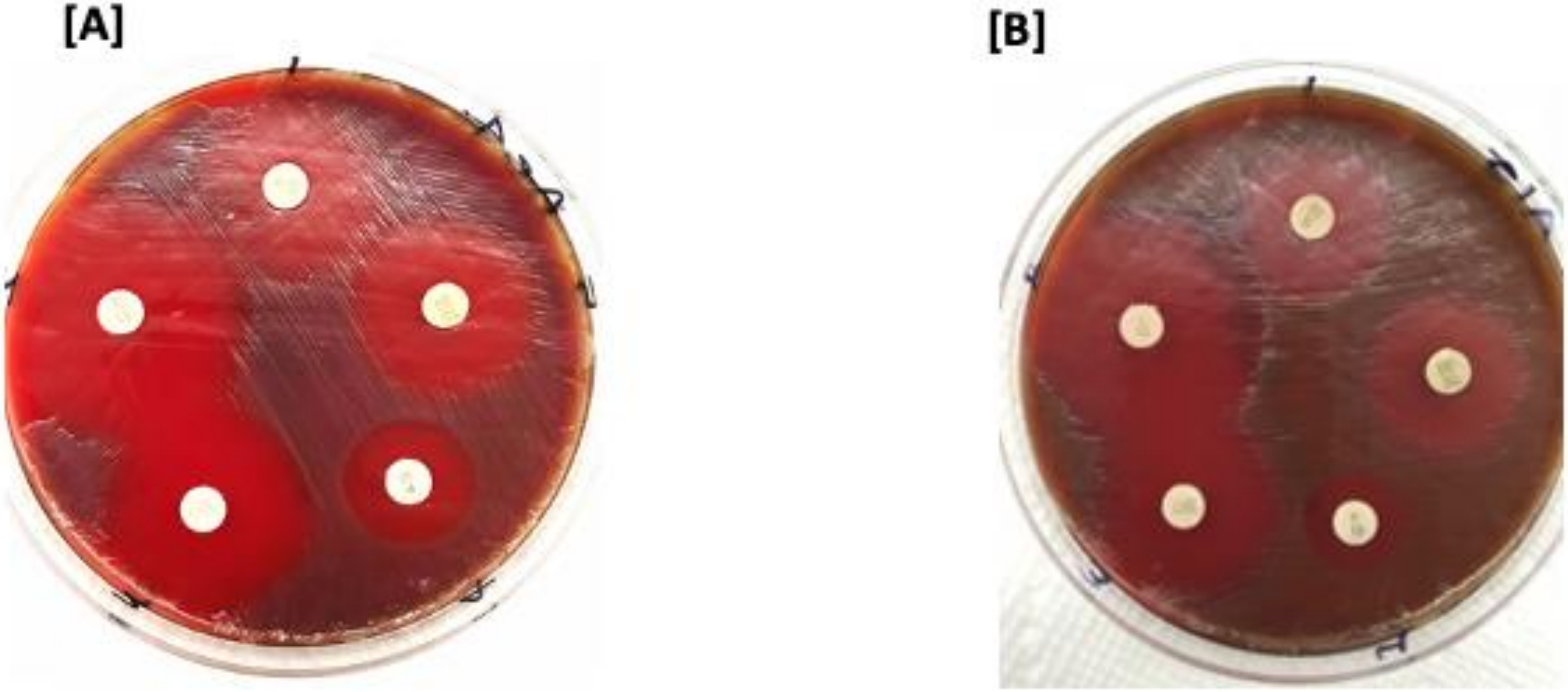

Fig.S1 Representative antibiotic disc diffusion assay for Sporosarcina cascadensis A4 (cascadiensis. L. masc. adj. cascadensis) [A], and Sporosarcina obsidiansis A15 (obsidiansis L. masc. adj. obsidiansis) [B]. The antibiotic discs are as listed from the top in order clockwise as streptomycin, erythromycin, penicillin, chloramphenicol, and tetracycline. Both isolates were sensitive to the assayed antibiotics. Methods: The A4 and A15 isolates were assayed by disc diffusion to streptomycin, erythromycin, penicillin, chloramphenicol, and tetracycline to determine antibiotic resistances as described in [29].
